# Barriers and facilitators affecting vasectomy acceptability (a multi stages study in a sample from north eastern of Iran), 2005-2007

**DOI:** 10.1186/1447-056X-10-5

**Published:** 2011-05-08

**Authors:** Afsaneh Keramat, Afsaneh Zarei, Masoumeh Arabi

**Affiliations:** 1 Reproductive Health Department, Shahroud University of Medical Sciences, Shahroud, Iran; 2 Student research committee, Shahroud University of Medical Sciences, Shahroud, Iran

**Keywords:** vasectomy, family planning, permanent method

## Abstract

**Background:**

In this study we aimed to find factors affecting vasectomy acceptability in Shahroud (north eastern Iran).

**Methods:**

This study was carried out in three stages. The first stage was a survey of couples that had the vasectomy procedure during 2004-2007 in the Shahroud area. In the second stage of the study we compared characteristics of the cases (the couples who had the vasectomy procedure during the study period) and controls (including couples with at least one child that choose other contraceptive methods excluding a vasectomy) using χ² and T student tests. In the third stage of the study we aimed to assess the knowledge and attitudes of those who did not choose to have a vasectomy as there contraception method by filling out questionnaires in personal interviews.

**Results:**

An increasing trend toward the vasectomy procedure was observed during 2005 to 2007. We found positive associations between male and female educational levels and choosing to have a vasectomy (p < 0.05). Majority of women (88.44%) thought that their husbands would prefer to have a tubectomy to a vasectomy.

**Conclusion:**

The study results show a necessity for both couples to participate in educational programs about the vasectomy procedure.

## Background

The vasectomy procedure is a safe, simple and permanent method of contraception and has a failure rate of less than 1%[1]. It is less expensive and equally as effective as female sterilization; however, vasectomies are one of the least used and least known methods of contraception throughout the world[2]. Worldwide, an estimated 33 million married women between 15 to 49 years old (less than 3%) rely on their partner's vasectomy for contraception [3].

Garcia Moren and co-workers suggested that greater spreading of information regarding the vasectomy procedure as a contraceptive method, greater links between male needs and the vasectomy procedure, and maintaining or increasing access to family planning[4].

In a nationwide practice-based survey in the United States conducted on 719 men receiving vasectomies, the researchers found that in spite of the variety of the U.S. population, vasectomy recipients are a homogeneous group [5].

Overall the prevalence of vasectomies is lower in developing countries. In Asia, with the exception of Bhutan, Iran, and the Republic of Korea, the occurrences of vasectomy has gradually declined over the past 15 years[6].

In the Islamic Republic of Iran, between 1993 and 2004, 500 training courses were conducted in public sector institutions. During the same period, an estimated 375,000 Iranians underwent the procedure, raising the prevalence of vasectomy from 0% to 3.5% in national contraception [7]. As it is mentioned in other reports, although the worldwide frequency of vasectomy is still much lower than tubectomy (3%-6% versus 28%), men are going to accept more responsibility for family planning [8].

In this study we aimed to find barriers and facilitators affecting vasectomy acceptability in Shahroud area (north eastern Iran) during 2005-2007.

## Materials and methods

This study that was approved by Research Committee of Shahroud Medical University, and was carried out in three stages. The first stage was a survey on couples that had a vasectomy during 2005-2007 in Shahroud area (in north east Iran). In this stage we assessed the number and characteristics of couples who chose this method and where they sourced their information about vasectomies from. Demographic characteristics that we assessed in this stage of study included age, education, occupation, age at marriage, number of child and the income of the couples. We have tried to find the association between the couples studied characteristics and their sources of information, and the number of couples that chose to have a vasectomy during the study period (2005-2007) using chi-square and t students' test statistical methods.

The second stage was a case-control study. The case group included couples who underwent the vasectomy procedure during the study period (all of the samples from first stage of study that lived in city area) and the study controls included couples with at least one child that received other contraceptive methods. The controls were selected randomly from a list of couples that underwent other contraceptive methods, except vasectomy, created from the centres' databases during the study period with same number of cases from each centre. Then we compared characteristics of case and controls using χ² and T student test.

In third stage of study we aimed to evaluate the knowledge and attitude of those who did not choose to have a vasectomy as their contraception method by interviewing women of the control group.

Data collected for this part of the study included filling in questionnaires through personal interviews. We informed the clients about the goals and the process of the study clearly in their own language (Persian). We have interviewed clients who agreed to participate; the consent was verbal in all of the health centres. The patients' identity was not revealed and patients' data was kept confidential.

## Results

In the first stage of the study we have found a total of 311 couples who underwent the vasectomy procedure during 2005 to 2007. The mean of the age of men and women were 39.5 ± 7.7 and 33.1 ± 6.5, respectively. As it is shown in table 1, 19.4% of men had more than 12 years of school education and 36.6% of them were educated between 5-9 school years. Among women 9.6% had more than 12 years of school education and 35.4% between 9 to 12 schooling years. Majority (85.5%) of vasectomy recipients were from urban areas. The trend towards vasectomy during the study period is shown in table 1. We have found a sharp increase in the numbers of couples who have chosen to have a vasectomy between 2005 and 2006. There were no significant associations between couples's demographic characteristics during the mentioned years. But we have found a significant difference in sources of information about vasectomy during 3 years (2005-2007) (table 1).

**Table 1:** Frequency of couples that chose vasectomy and their demographic characteristics and sources of information during 2005-2007

year	2005	2006	2007	total	significance
Number of vasectomy's	16	145	155	311	
Male age					NS*
(mean)	38.4 ± 4.8	39.2 ± 7.3	39.9 ± 8.2	39.5 ± 7.7	
Female age					NS*
(mean)	32 ± 6.9	33.2 ± 6.9	33.06 ± 6	33.1 ± 6.5	
Age of marriage					
(mean)	19 ± 4.1	21.8 ± 4.6	24.5 ± 9.4	22.9 ± 7.5	P < 0.05
Number of child	2.7 ± 1.1	2.6 ± 1.1	2.8 ± 1.2		NS*
**Male education**					NS*
No education	1(6.3)	6(4.1)	4(2.6)	11(3.4)	
Less than 5 yrs	7(43.7)	32(21.6)	24(15.4)	64(19.7)	
5-9	4(25)	49(33.1)	64(41/)	117(36.6)	
9-12	3(18/7)	31(20/9)	33(21.2)	67(20.9)	
more than 12	1(6/3)	30(20/3)	31(19.4)	62(19.4)	

**Female education**					
No education	0(0)	3(2.1)	3(1.9)	6(1.9)	NS*
Less than 5 yrs	5(33.3)	43(29.9)	28(18.1)	76(24.2)	
5-9	5(33.3)	32(22.2)	54(34.8)	91(29)	
9-12	5(33.3)	51(35.4)	55(35.5)	111(35.4)	
more than 12	0(0)	15(10.4)	15(9.7)	30(9.6)	

**Citizen**					
Urban	14(87.5)	127(84.7)	130(86.7)	271(85.5)	NS*
Rural	2(12.5)	23(15.3)	20(13.3)	45(14.2)	

**Source of information**					
Media	0(0)	2(1.4)	9(6.5)	11(3.6)	P < 0.05
Health workers	14(87)	117(79.1)	74(53.6)	205(67.9)	
Friends and families	2(12.5)	27(18.2)	51(37)	80(26.5)	
others	0	2(1.4)	4(2.9)	6(2)	

* Non significant

In the second stage of the study we compared characteristics of couples (gathered from the first stage) who accepted the vasectomy procedure (case group), with couples that chose other contraceptive methods (control group). There were significant relationships between male and female educational level and choosing to have a vasectomy (p < 0.05).

The level of education was significantly higher in the vasectomy group as compared with those who had chosen tubectomy among both males and females (p < 0.05) (table 2). Vasectomy receiving couples also had lower age and lower child numbers and higher income than couples who chose tubectomy. Even after adjustment for child number and age, comparison of vasectomy group with couples that had chosen non permanent methods revealed that the education level in the vasectomy group was significantly lower (p < 0.05).

**Table 2:** frequency of study population (Second stage) divided by their contraception method, demographic characteristics and sources of information

Case and control	Vasectomy group(cases)	non permanent contraception methods	Tubectomy	Total	Significant
Number of vasectomy's	269	168	43	480	
Male age					
(mean)	39.6 ± 7.3	38.8 ± 7	46.1 ± 7.4	39.9 ± 7.5	P < 0.05
Female age					
(mean)	33.03 ± 6.3	33.8 ± 6.1	45.2 ± 5.9	33.9 ± 6.5	P < 0.05
Age of marriage					
(mean)	23.18 ± 6.6	19.42 ± 3.7	18.55 ± 3.7	21.44 ± 5.8	P < 0.05
Number of child	2.63 ± 1.03	2.3 ± 0.65	3.6 ± 1.1	2.6 ± 0.98	P < 0.05

**male education**	n = 272	n = 166	n = 42	n = 480	
No education	4(1.5)	1(0.6)	4(9.6)	9(1.9)	P < 0.05
Less than 5 yrs	46(16.9)	28(16.6)	17(40.5)	91(19)	
5-9	100(36.8)	35(21.1)	9(21.4)	144(30)	
9-12	65(23.9)	47(28.2)	7(16.7)	119(24.8)	
more than 12	57(21)	55(33.1)	5(11.9)	117(24.4)	

**Female education**	n = 267	n = 168	n = 43	n = 478	
No education	3(1.1)	2(1.2)	3(7)	8(1.7)	P < 0.05
Less than 5 yrs	54(20.2)	38(22.6)	22(51.2)	114(23.8)	
5-9	82(30.7)	33(19.6)	5(11.6)	120(25.1)	
9-12	101(37.8)	62(36.9)	10(23.3)	173(36.1)	
more than 12	27(10.1)	33(19.6)	3(7)	63(13.2)	

**Source of information**	n = 258	n = 142	n = 43	n = 444	
Media	11(4.3)	1(0.7)	1(2.3)	14	P < 0.05
Health workers	165(64)	120(84.5)	23(53.5)	308	
Friends and families	75(29.1)	5(3.5)	1(2.3)	81	
other	7(2.7)	16(11.3)	18(41.9)	41	

In third stage of the study we evaluated that the knowledge and attitude of those who did not choose vasectomy as a contraception method by interviewing women of the control group of stage 2 (table 3). There was no significant association between the knowledge and attitude about vasectomies as a kind of contraception. The higher and lower knowledge and attitude scores belong to the users of injectable methods (such as Depot medroxyprogesterone acetate) and condom, respectively. We also have found that there is no significant difference in knowledge and attitude about vasectomy between tubectomy and non permanent contraception receivers. Couples who have chosen tubectomy had significantly higher mean of age and child number compared with non permanent method users (p < 0.05).

**Table 3:** frequency of study population (Third stage) in based on their attitude toward vasectomy

Attitude	Extremely disagree	Disagree	No idea	Agree	Extremely agree
	N (%)	N (%)	N (%)	N (%)	N (%)
I prefer tubectomy to vasectomy	73(30.67)	85(35.71)	8(3.36)	48(21.16)	24(10.08)
I do not agree with permanent Contraception	41(17.29)	48(20.25)	6(2.53)	82(34.59)	60(25.31)
Impotency is a side effect of vasectomy	28(11.76)	75(31.51)	105(44.1)	25(10.50)	5(2.1)
The side effects from having a vasectomy are greater than having a tubectomy	75(31.51)	120(50.42)	21(8.82)	15(6.3)	7(2.94)
If we decide to have a vasectomy I prefer not to tell anyone	57(23.75)	87(36.25)	12(5)	52(21.66)	32(13.33)
I would choose to have a vasectomy only in if having a tubectomy was not possible	61(25.52)	107(44.76)	15(5.17)	42(17.57)	14(5.85)
My family are opposed to vasectomy's	65(10.12)	112(47.25)	7(2.95)	39(16.45)	14(5.9)
I have suggested to male family members to consider having a vasectomy	24(10)	43(18)	23(9.6)	75(31.2)	75(31.2)
My husband thinks that one of the side effects of having a vasectomy is impotency	9(3.8)	42(15.57)	150(62.76)	31(12.97)	7(2.92)
My husband would prefer to have a tubectomy then a vasectomy	3(1.26)	23(9.66)	11(4.62)	116(48.73)	85(35.71)
My husband's family disagrees with tubectomy	24(10)	43(17.91)	23(9.58)	75(31.25)	75(31.25)

Assessment of the attitude towards vasectomy among women that were using contraceptive methods other vasectomy, displayed that majority of them did not agree with a permanent method (table 3). A majority (88.44%) of the women who have chosen contraceptives other than vasectomy assume that their husbands would prefer tubectomy to vasectomy. Sixty two percent of these women did not know if their husbands are concerned about impotency. Thirty five percent of interviewed women said that if they choose vasectomy they would prefer to keep it a secret.

## Discussion

Worldwide, approximately 3-6% of couples are using vasectomy as a method of contraception [3,9]. In the first stage of study we have found total 311 couples that underwent vasectomy during 2005 to 2007. The population in Shahroud area is about 200,000, which means there are 62229 couples. It means that prevalence of vasectomy in Shahroud area is approximately 5% that is similar to vasectomy's worldwide prevalence. Other studies also report the same occurrences in Iran. In this study we have found a sharp increase in numbers of couples who have chosen vasectomy between 2005 and 2006; however, there was no difference between 2006 and 2007. By comparison, the vasectomy prevalence has progressively declined in Asia over the past 15 years with the exception of Bhutan, Iran and the Republic of Korea [6].

As It is displayed in table1, 19.4% of men had more than 12 years of school education and 36.6% of males that accepted vasectomy had between 5-9 of school education. Among women 9.6% were educated more than 12 years and 35.4% of females whom their husbands accepted vasectomy had between 9 to 12 years of school education. A majority (85.5%) of vasectomy recipients were from urban area. Barone, M.A. and co-workers in their nationwide practice-based survey, have founded that Low-income, minority and less educated men were underrepresented among vasectomy recipients [5]. But in a survey of male subjects attending the Kingston Contraceptive Clinic for vasectomy, showed that usage of vasectomy was predominantly related to social class [10]. Some other studies report that those who choose vasectomy are belong to higher socioeconomic group [11]. A study in Nepal found that the major occupation of vasectomised men was agriculture (73.7%), with a literacy rate of 83.0% [12]. We have found a significant difference in sources of information about vasectomy during 3 years (2005-2007) (table1). Although the most important sources of information about vasectomy in all 3 years were health providers, the role of health workers in acceptance of vasectomy was lower and the role of media and friends were higher in 2007 as compared with previous years. Similarly in other studies, the most important sources of information about vasectomy were health providers followed by family or friends [5]. The result of present study shows the necessity of carrying out the educational programs for improving the knowledge and attitude of the health workers.

Level of education was significantly higher in vasectomy group among both male and female (table2). Education level in vasectomy group was significantly lower than people that have chosen non permanent methods (p < 0.05).

In our study, couples who have accepted vasectomy were significantly more educated than couples that have accepted tubectomy. They also had lower age, lower child number and higher income. Therefore we suggest specific consultations and education programs for people who have indications for choosing permanent methods of contraception.

Vasectomy is more common than female sterilization in only 5 countries. These countries are Bhutan, Denmark, the Netherlands, New Zealand and Great Britain. In 8 countries: Australia, Bhutan, Canada, the Netherlands, New Zealand, the Republic of Korea, Great Britain and the United States, the prevalence of vasectomy use exceed 10%. New Zealand has the highest occurrence of vasectomy at 19.3 [13]. Although one of the most important reasons mentioned by couples who choose to have a vasectomy is the lower cost; [14,15] however, in our study the higher socioeconomic level of people who accepted vasectomy as compared with tubectomy group shows that the mentioned factor does not have an important role in our area. Other causative factors to selection of vasectomy are lower side effects, mortality and simplicity of the surgical procedure of vasectomy [16,17]

In a cross-sectional study carried out in Iran, one of the most important reasons for vasectomy refusal was concern about complications, including the risk of sexual disability after vasectomy [18].

A descriptive study that was conducted in south-western Ethiopia displayed that men's choice of vasectomy as a method of contraception was 79%. The prevalence of men that were not in agreement with vasectomy because of possible loss of children due to death or divorce was 21%" [19].

In the third stage of present study after assessment the attitude about vasectomy only 12.6% of women were worried about impotency and the majority of the women who have chosen contraceptives, except vasectomy, thought that their husbands would prefer tubectomy to vasectomy. Therefore we suggest a consultation and specific educational programs for both males and females. Similar findings were reported in several previous studies [20-22].

Culture and community aspects influence the ability and willingness of men to obtain a vasectomy. In our study 35 percent of interviewed women said that they would prefer concealment about their contraception method if they choose vasectomy.

## Conclusion

There was an increasing trend towards acceptance of vasectomy during the study period. The results of the study show an acceptable level of knowledge about vasectomy among users of other contraceptive methods. Iran's policy in requiring both men and women to take a class on modern contraception before receiving a marriage license and increasing knowledge about vasectomy might be important facilitators that can influence vasectomy acceptance. In our study couples that accepted the vasectomy procedure were significantly more educated than couples that accepted tubectomy, but they were significantly lower in education level compared with users of non permanent methods. For women, one of the most important barriers to acceptance of vasectomy is the negative attitude of men towards vasectomy.

Various strategies should be implemented that aim to increase vasectomy use:

1. Specific consultations for couples who accepted a permanent method especially tubectomy.

2. The necessity of both couples participation in consultation sessions targeting men.

3. Vasectomy use can also be increased through targeting the staff and clinics at healthcare centres. Additional training should be offered for staff in order to create competent, committed staff who can effectively communicate with men. The staff members should encourage vasectomy.

## Competing interests

The authors declare that they have no competing interests.

## Authors' contributions

AK contributed to the study, design, analysis and acquisition of data to complete the final report. AZ and MA contributed study design and data collection, all authors approved the manuscript.
